# Supplementary material for: FAM83D directs protein kinase CK1α to the mitotic spindle for proper spindle positioning
Source: EMBO Rep. 2019 Jul 24;20(9):e47495. doi: 10.15252/embr.201847495 (PMC6726907; doi:10.15252/embr.201847495)
Supplement: Supplementary file 1 — Appendix [file EMBR-20-e47495-s001.pdf]

FAM83D directs protein kinase CK1α to the mitotic spindle for proper spindle positioning

Luke J. Fulcher[1], Zhengcheng He[2], Lin Mei[2], Thomas J. Macartney[1], Nicola T. Wood[1], Alan R. Prescott[3], Arlene J. Whigham[4], Joby Varghese[1], Robert Gourlay[1], Graeme Ball[3], Rosemary Clarke[4], David G. Campbell[1], Christopher A. Maxwell[2], and Gopal P. Sapkota[1*].

[1]Medical Research Council, Protein Phosphorylation and Ubiquitylation Unit, University of Dundee, Dundee, United Kingdom. [2]Michael Cuccione Childhood Cancer Research Program, British Columbia Children's Hospital, University of British Columbia, Vancouver, Canada.[3]Dundee Imaging Facility, School of Life Sciences, University of Dundee. [4]Flow Cytometry and Sorting Facility, School of Life Sciences, University of Dundee.

* Correspondence and requests for materials should be addressed to: g.sapkota@dundee.ac.uk

List of Appendix Figure Legends, Figures and Files:

Appendix Figure S1 legend
Appendix Figure S2 legend
Appendix Figure S1
Appendix Figure S2
Appendix File S1

Appendix Figure S1: CK1α knockdown phenocopies the effect of FAM83D knockout on spindle mispositioning:

A: Western blot analysis of U2OS cell extracts 48 h after treatment with scrambled siRNA (siScramble) controls or siRNA targeting CK1α (siCK1α). **B:** Representative images of mitotic U2OS cells stained with Hoechst, beginning at metaphase and taken every 5 min as they progressed through division. Mitotic stage was determined by chromosome condensation and is indicated by the coloured boxes. Scale bar, 20 µm. **C:** Graphical representation of the kinetics of transition from metaphase alignment (yellow) to anaphase (green), and cytokinesis (blue), determined by the morphology of chromosomes and daughter cells, respectively. The kinetics of 100 mitotic cells per genotype are plotted as measured for 50 cells per experiment from 2 independent experiments. **D:** Length of time needed to transition from metaphase to anaphase. Mean ± SEM is plotted for 2 independent experiments, which each measured 50 mitotic cells per genotype (n = 100 mitotic cells per genotype total). *** $p < 0.0001$, Student's *T*-test. **E:** Representative images of mitotic U2OS cells stained with Hoechst and grown on L-shaped micropatterns previously coated with fibronectin. The cell division angle at anaphase is indicated (yellow line). Scale bar, 20 µm. **F:** Circular graphs, superimposed on a L-shaped micropattern, show the distribution of cell division angles measured at anaphase. Angles are plotted for 100 U2OS cells per genotype measured from 2 independent experiments. The percentages of division angles±15° from the expected axis (red line) are indicated. **G:** Heatmap additive intensities of RFP-actin localization in two representative mitotic U2OS cells for each genotype grown on fibronectin-coated, L-shaped micropatterns. Arrowheads indicate polarized cortical actin.

Appendix Figure S2: Disruption of the FAM83D-CK1α interaction promotes asymmetric membrane elongation but does not affect daughter cell size.

A: Representative bright field images initiating at metaphase for mitotic U2OS cells taken every 1 min as they progress through division. Asymmetric membrane elongation, or membrane blebbing, is indicated by red arrowheads and only occurred on one daughter cell. Scale bar, 20 µm. **B**: Percentage of mitotic cells that displayed membrane blebbing. Mean of 50 cells per genotype are plotted from 2 independent experiments. Error bars; SEM. * $P < 0.005$, ANOVA. **C**: Daughter cell size ratio following U2OS cell division remains unchanged. Mean of 50 cells per genotype are plotted from 2 independent experiments. Error bars; SEM.

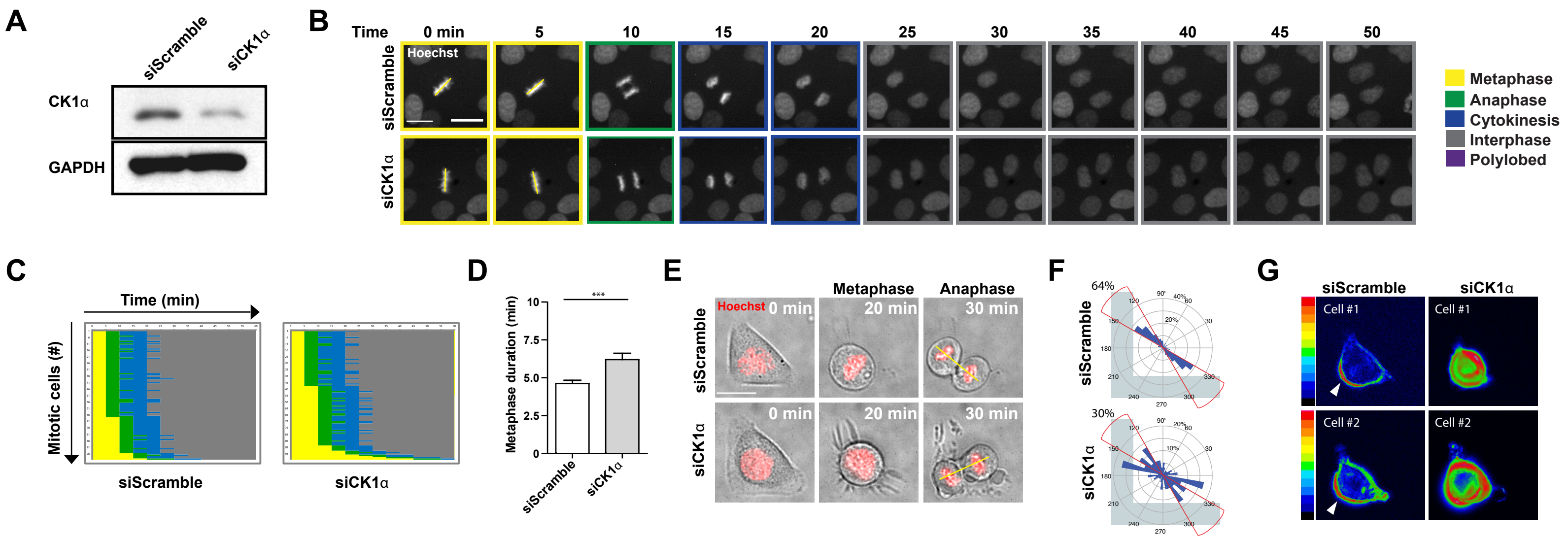
A
siScramble
siCK1α
CK1α
GAPDH
B
Time
0 min
5
10
15
20
25
30
35
40
45
50
Hoechst
siScramble
siCK1α
Metaphase
Anaphase
Cytokinesis
Interphase
Polylobed
C
Time (min)
Mitotic cells (#)
siScramble
siCK1α
D
Metaphase duration (min)
10
7.5
5.0
2.5
0
***
siScramble
siCK1α
E
Metaphase
Anaphase
Hoechst
0 min
20 min
30 min
siScramble
siCK1α
F
64%
30%
siScramble
siCK1α
G
siScramble
siCK1α
Cell #1
Cell #2

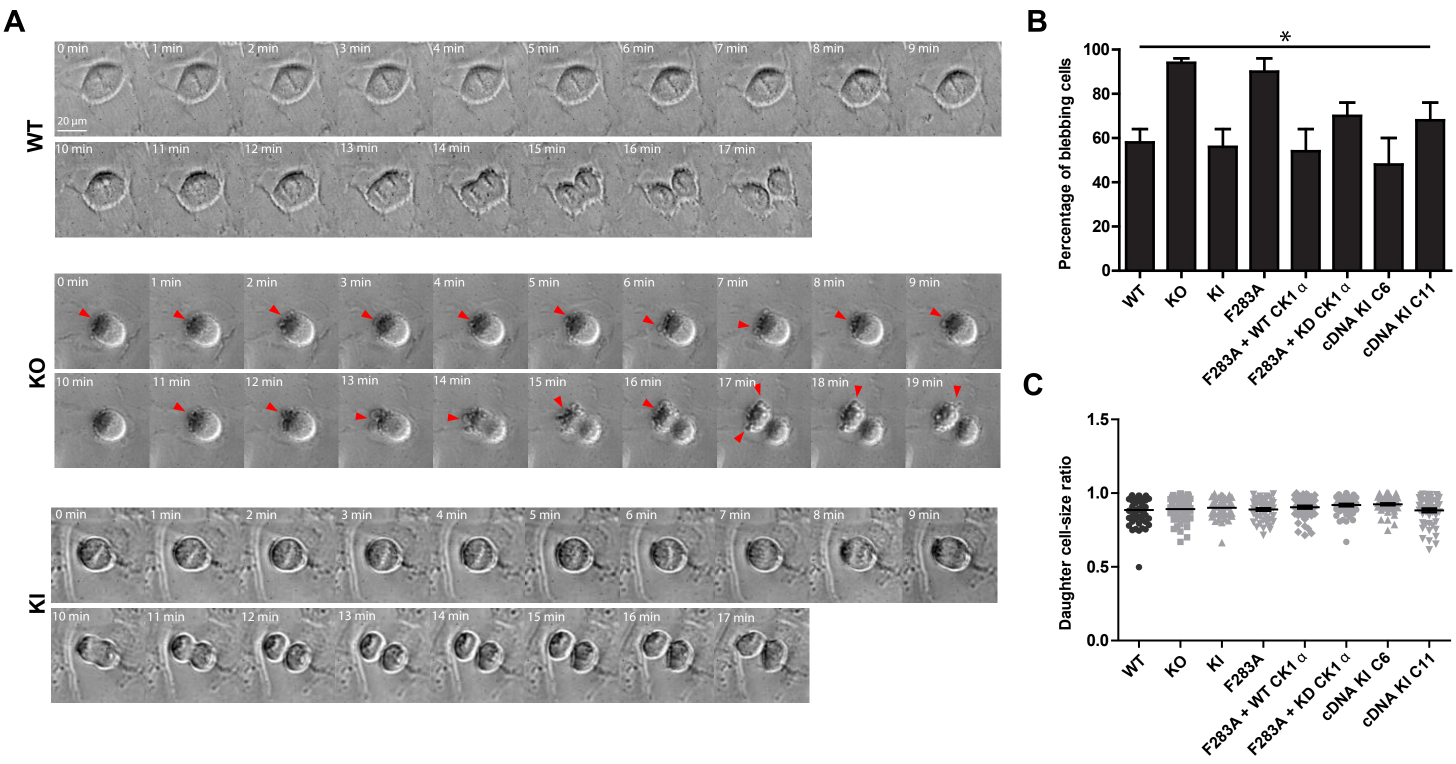

Appendix Figure S2

Appendix File S1: The following ImageJ macro was developed in order to measure mean CK1α staining intensity in the spindle.

```
// Spindle stain quantification macro
// - measure stain in spindle region bounded by DAPI ring exterior
// - measure stain outside DAPI ring region to estimate background
// - for each nucleus identified, report ratio spindle/background
//
// for Luke Fulcher, 2018
//
// Copyright Graeme Ball 2018, Dundee Imaging Facility
// License: Creative Commons CC-BY-NC-SA
//

// parameters
chDAPI = 3;  // channel number for DAPI
chCK1a = 1;  // channel for CK1alpha stain
minSizeSpindle = 8000;  // minimum number of pixels in a spindle
region
Dialog.create("Measure_spindle_ratio");
Dialog.addNumber("DAPI channel", chDAPI);
Dialog.addNumber("CK1a channel", chCK1a);
Dialog.show();
chDAPI = Dialog.getNumber();
chCK1a = Dialog.getNumber();

roiManager("reset");
roiManager("UseNames", "true");
roiManager("Show All with labels");
run("Set Measurements...", "area mean standard min integrated
display redirect=None decimal=3");
setOption("BlackBackground", true);

// find spindle regions
Stack.setDisplayMode("grayscale");
Stack.setChannel(chDAPI);
run("Duplicate...", " ");
run("Convert to Mask", "method=Otsu background=Dark calculate
black");
run("Fill Holes");
run("Analyze Particles...", "size=" + minSizeSpindle + "-Infinity
exclude add");
close();

// create CK1a whole-cell mask with separated cells
Stack.setChannel(chCK1a);
run("Duplicate...", " ");
setAutoThreshold("Triangle dark");
run("Convert to Mask");
run("Fill Holes");
run("Median...", "radius=15");  // clean up rough edges
run("Make Binary");
run("Watershed");
```

```
// for each spindle ROI, attempt to find containing cell ROI and
create cytoplasm ring ROI
nNuclei = roiManager("count");
setThreshold(1, 255);
for (i = 0; i < nNuclei; i++) {
        roiManager("select", i);
        roiManager("rename", "spindle" + i);
        getSelectionBounds(x, y, w, h);
        x = round(x + w/2);
        y = round(y + h/2);
        doWand(x, y);
        roiManager("add");  // cytoplasm region centred on this
spindle
        newCellIndex = roiManager("count") - 1;
        indices = newArray(i, newCellIndex);
        roiManager("select", indices);
        roiManager("XOR");
        roiManager("add");
        newCytoIndex = roiManager("count") - 1;
        roiManager("select", newCytoIndex);
        roiManager("rename", "cytoplasm" + i);
        roiManager("select", newCellIndex);
        roiManager("delete");
}
close(); // close CK1a whole-cell mask

// make measurements using paired ROIs and write to Results table
run("Clear Results");
Stack.setChannel(chCK1a);
for (i = 0; i < nNuclei; i++) {
        row = nResults;
        setResult("cellID", row, i);
        roiManager("select", i);
        getRawStatistics(nPixels, spMean, min, max, std);
        setResult("spindleMean", row, spMean);
        setResult("spindleTotal", row, (spMean * nPixels));
        roiManager("select", i + nNuclei);  // corresponding
cytoplasm ROI
        getRawStatistics(nPixels, cyMean, min, max, std);
        setResult("cytoMean", row, cyMean);
        setResult("ratioS/C", row, (spMean/cyMean));
}

//run("From ROI Manager");
```
