# Supplementary material for: FAM83D directs protein kinase CK1α to the mitotic spindle for proper spindle positioning
Source: EMBO Rep. 2019 Jul 24;20(9):e47495. doi: 10.15252/embr.201847495 (PMC6726907; doi:10.15252/embr.201847495)
Supplement: Supplementary file 2 — Expanded View Figures PDF [file EMBR-20-e47495-s002.pdf]

Expanded View Figures

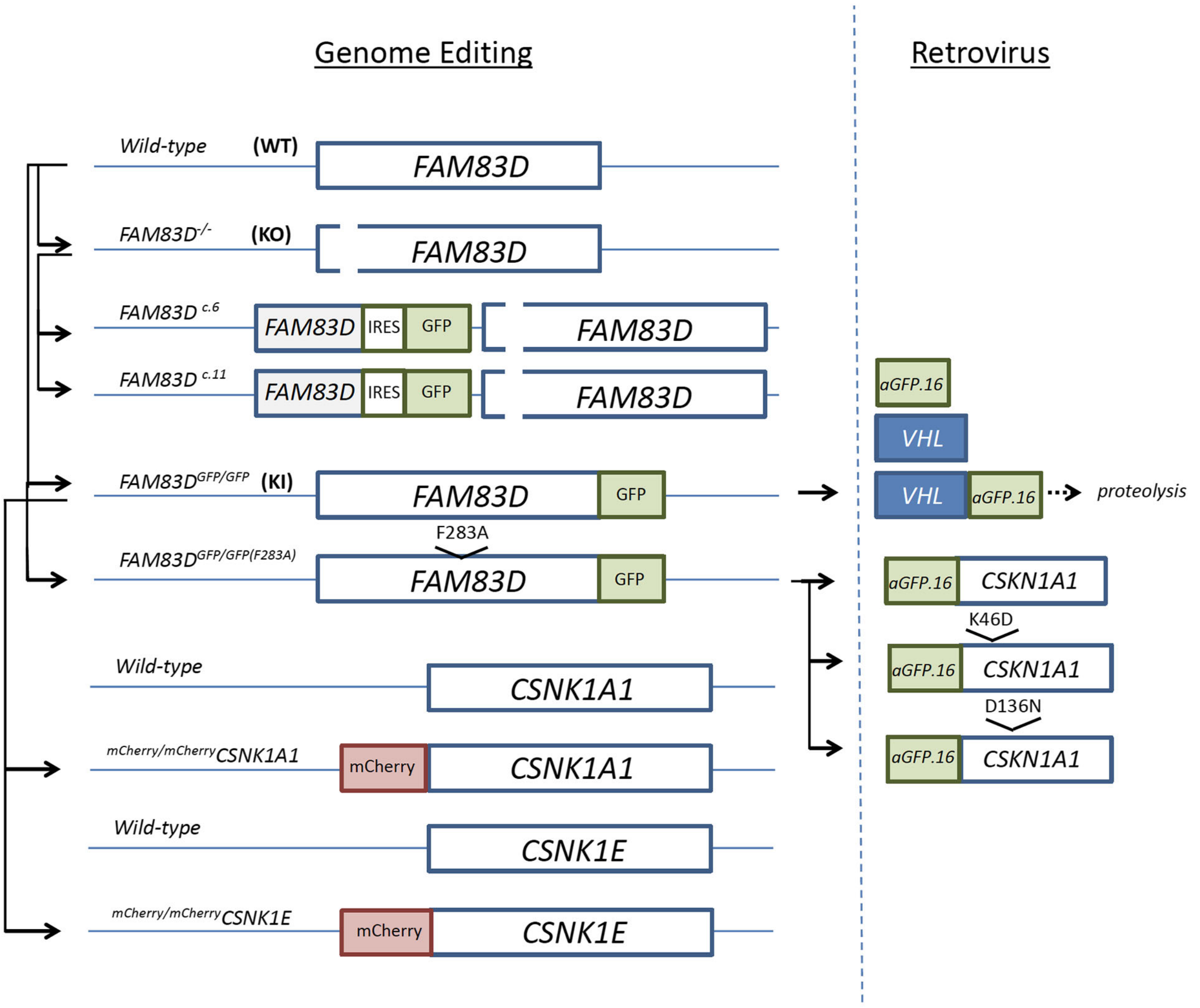

Figure EV1. Schematic of the CRISPR/Cas9 gene editing strategies, and retrovirally expressed nanobody-based systems used in this study.

Schematic detailing the CRISPR/Cas9 gene editing strategies employed to generate the indicated cell lines (left-hand side). The schematic on the right-hand side details the retrovirally expressed nanobody-based degradation (VHL-aGFP.16), and targeting (aGFP.16-CK1) strategies used in this study.

 © 2019 The Authors

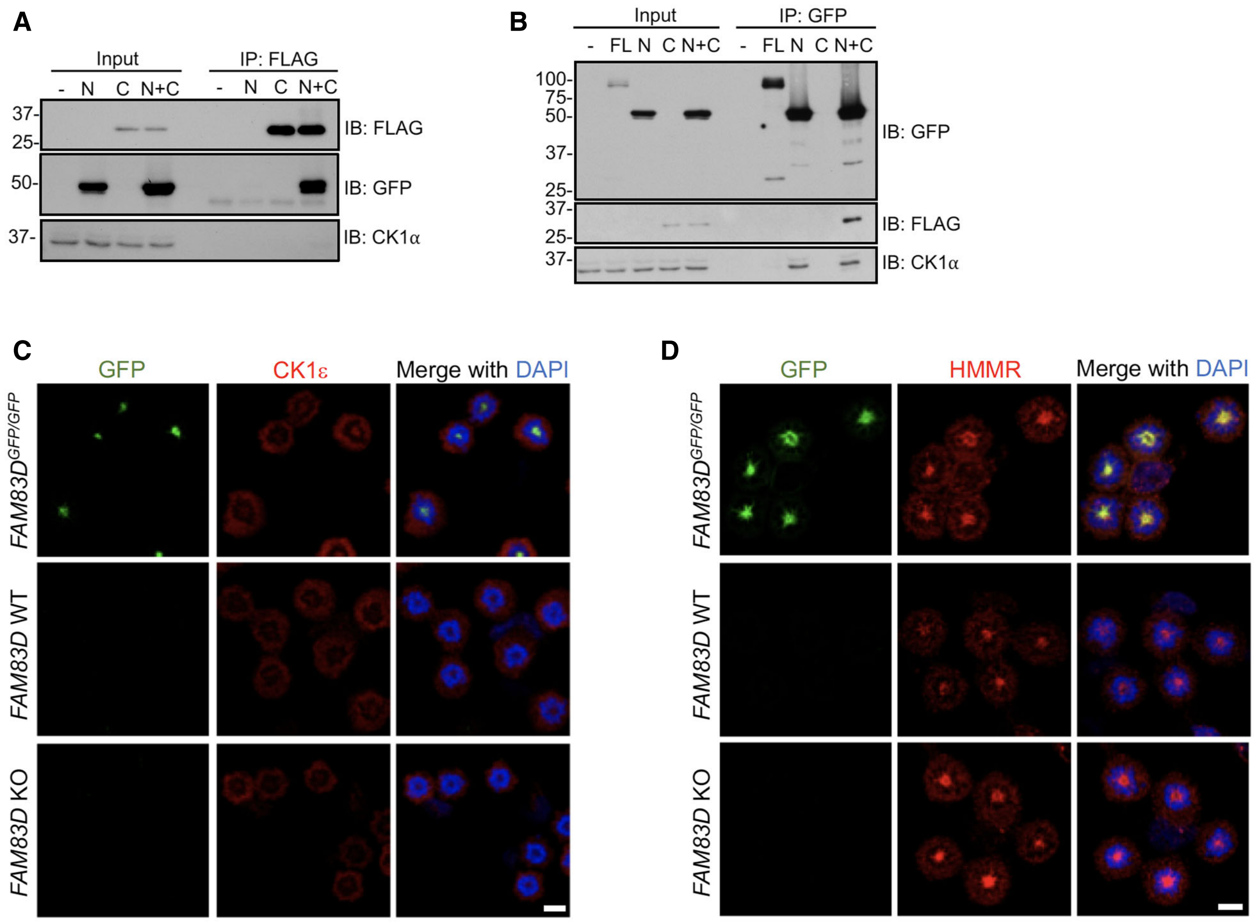

Figure EV2. Exploring the mechanism and specificity of the FAM83D–CK1 interaction.

A, B *FAM83D*$^{-/-}$ U2OS cells were transiently transfected with plasmids encoding GFP-tagged FAM83D N-terminus incorporating the DUF1669 (N), FLAG-tagged FAM83D C-terminus (C) domain lacking the DUF1669, or with both N and C fragments together; as an additional control, full-length (FL) FAM83D was also included in panel (B). Cells were lysed and extracts subjected to anti-FLAG (A) or anti-GFP (B) immunoprecipitations (IP). Whole-cell extracts (input) and IP samples were separated by SDS–PAGE, before immunoblotting (IB) with the indicated antibodies.

C, D Wild-type (WT), *FAM83D*$^{-/-}$ knockout (KO) and *FAM83D*$^{GFP/GFP}$ knockin U2OS cells were synchronised in mitosis with STLC, before being subjected to anti-CK1ε (C) or anti-HMMR (D) immunofluorescence and GFP fluorescence microscopy. DNA is stained with DAPI. Scale bars, 20 µm.

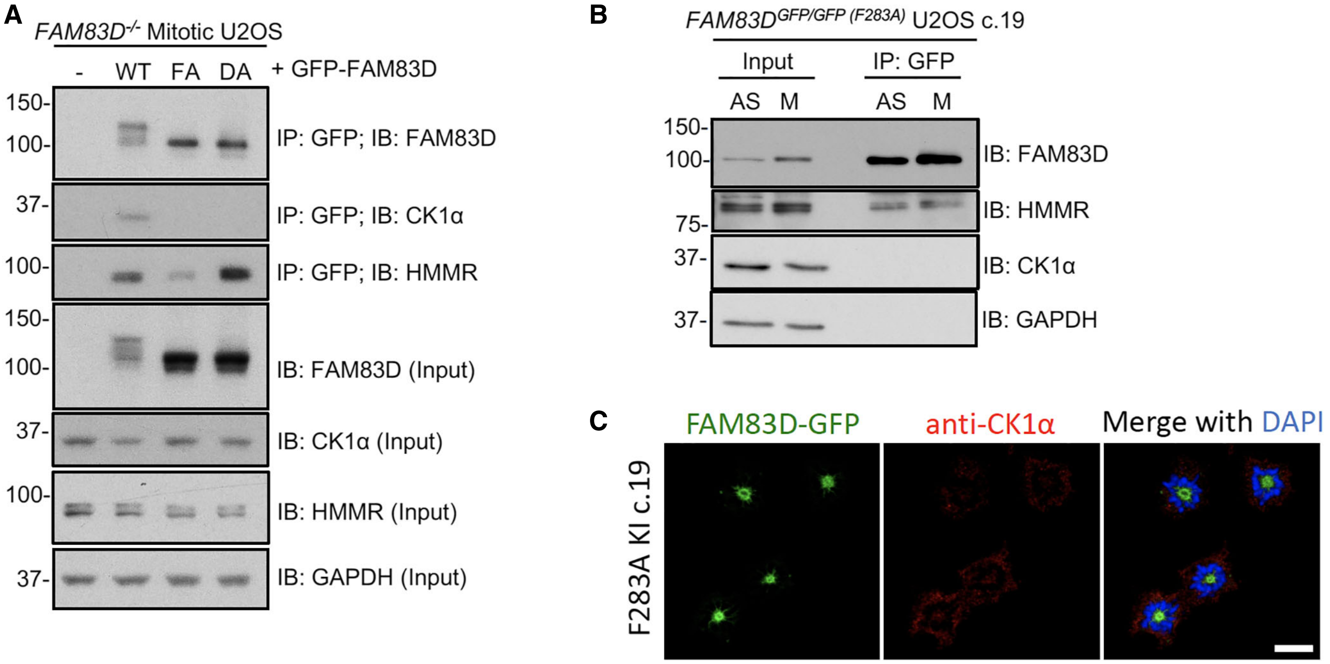

Figure EV3. Testing CK1-binding-deficient FAM83D mutants in mitosis.

A *FAM83D*$^{-/-}$ knockout (KO) U2OS cells were transiently transfected with vectors encoding GFP-FAM83D (WT), GFP-FAM83D(F283A) (FA) or GFP-FAM83D(D249A) (DA). Untransfected cells were included as a control (–). Following transfection, cells were synchronised in mitosis with STLC. Mitotic cells were collected, lysed and subjected to anti-GFP immunoprecipitation (IP) with GFP TRAP beads. Whole-cell extracts (input) and IP samples were immunoblotted (IB) with the indicated antibodies.

B *FAM8D*$^{GFP/GFP(F283A)}$ knockin U2OS cells (clone 19) were synchronised in mitosis with STLC (M). Asynchronous (AS) cells were included as a control. Cells were lysed and subjected to anti-GFP IP with GFP TRAP beads. Whole-cell extracts (input) and IP samples were IB with the indicated antibodies.

C The cell line described in (B) was subjected to anti-CK1α immunofluorescence and GFP fluorescence microscopy. DNA is stained with DAPI. Scale bars, 20 μm.

 © 2019 The Authors

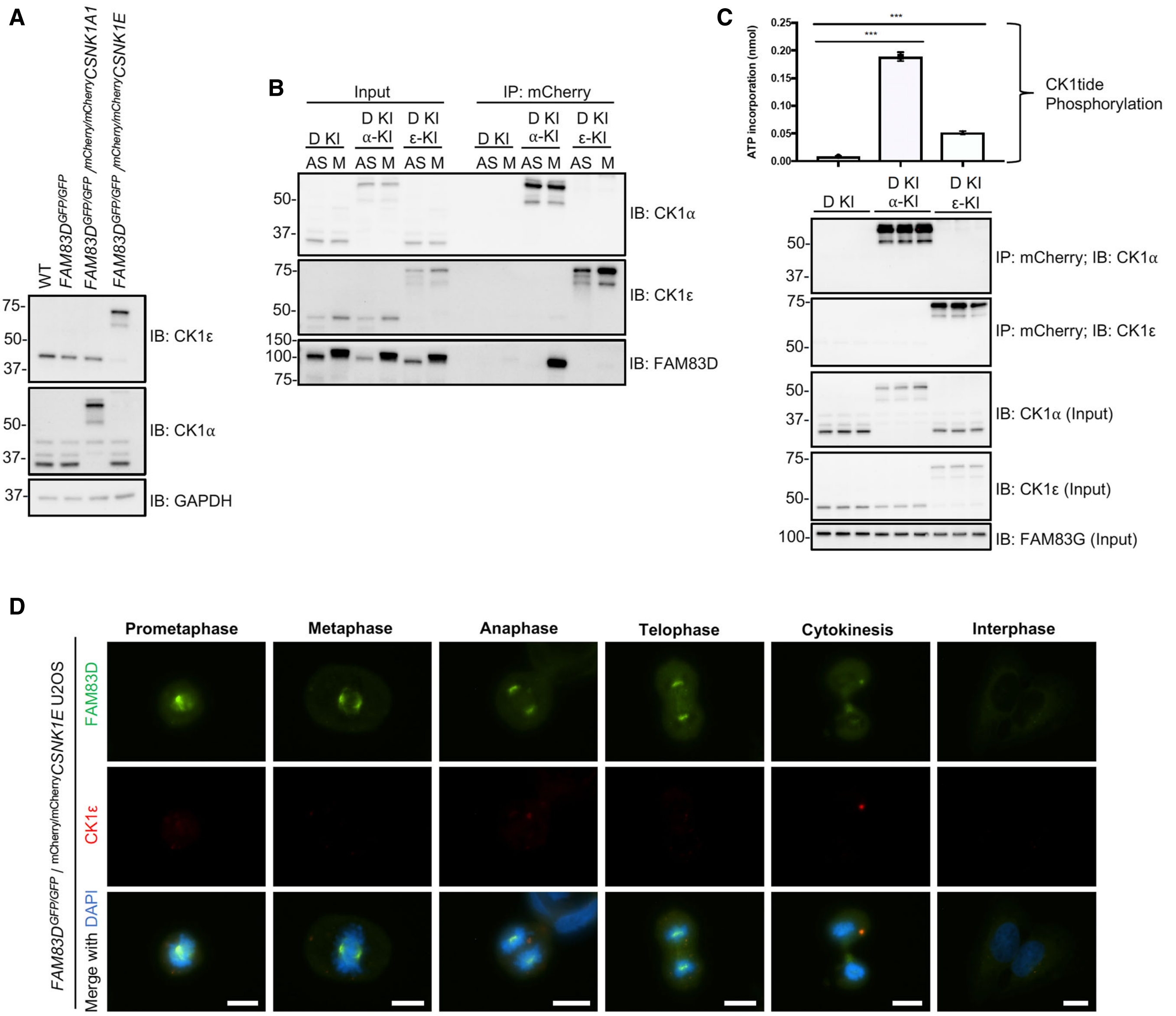

Figure EV4. Verification of $^{mCherry/mCherry}$*CSNK1A1* and $^{mCherry/mCherry}$*CSNK1E* U2OS knockin cells.

A Wild-type (WT), *FAM83D*$^{GFP/GFP}$ knockin (D-KI), *FAM83D*$^{GFP/GFP}$ knockin with $^{mCherry/mCherry}$*CSNK1A1* (D-KI, α-KI) and *FAM83D*$^{GFP/GFP}$ knockin with $^{mCherry/mCherry}$*CSNK1E* (D-KI, ε-KI) U2OS cells were lysed and subjected to immunoblotting (IB) with the indicated antibodies.

B The cell lines described in (A) were synchronised in mitosis with STLC (M) or left asynchronous (AS). Cells were lysed and subjected to immunoprecipitation (IP) with RFP TRAP beads, before immunoblotting (IB) with the indicated antibodies.

C The cell lines described in (A) were lysed and subjected to IP with RFP TRAP beads, and subjected to an *in vitro* [γ^{32}P]-ATP kinase assay using an optimised CK1 substrate peptide (CK1tide) and radiolabeled ATP. n = 3, Error bars, SEM, ***P < 0.0001; ANOVA.

D Asynchronous *FAM83D*$^{GFP/GFP}$/$^{mCherry/mCherry}$*CSNK1E* knockin U2OS cells were fixed and imaged. Representative images from the indicated cell cycle stages are included. Scale bar, 10 µm.

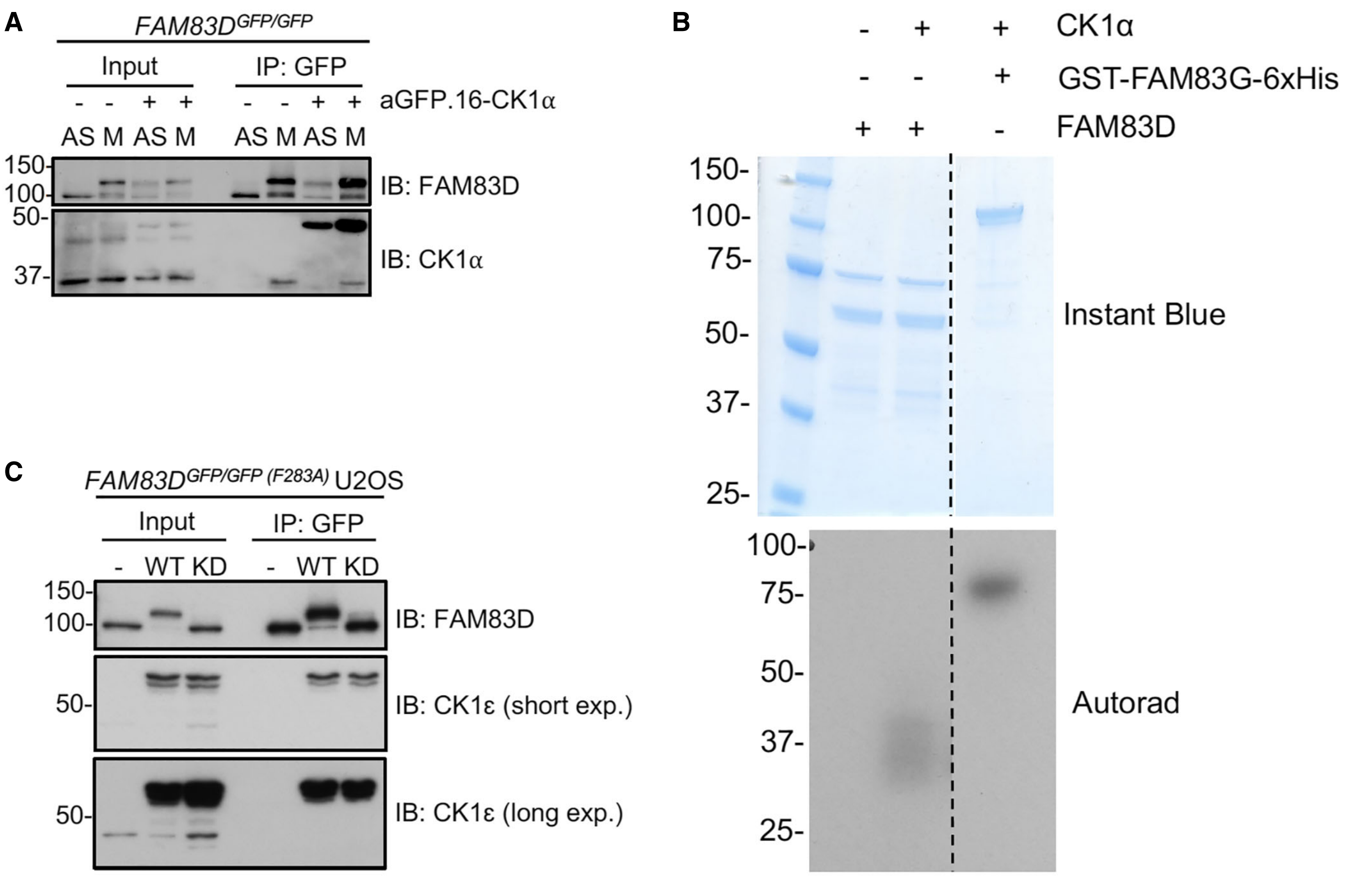

Figure EV5. CK1α does not appear to phosphorylate FAM83D *in vitro*.

A STLC-synchronised mitotic (M) *FAM83D*$^{GFP/GFP}$ knockin cells and *FAM83D*$^{GFP/GFP}$ knockin cells stably expressing aGFP.16-CK1α were subjected to GFP TRAP immunoprecipitation (IP), and extract (input) and IP samples were subjected to immunoblotting (IB) with the indicated antibodies. Asynchronous (AS) cells were used as controls.

B An in vitro kinase assay was set up using recombinant GST-FAM83D and GST-CK1α. GST-FAM83D without CK1α is a negative control. Recombinant GST-FAM83G-6xHis serves as a positive control for CK1α activity. Following incubation with radioactive ATP, reactions were stopped and subjected to SDS–PAGE. The gel was stained with InstantBlue and imaged, before being subjected to autoradiography (autorad).

C *FAM83D*$^{GFP/GFP(F283A)}$ cells were infected with retroviruses encoding wild-type aGFP.16-CK1ε (WT), or a kinase-dead aGFP.16-CK1ε (D128N) mutant (KD). Uninfected cells (–) were included as a control. Cells were lysed and subjected to anti-GFP IP and IB with the indicated antibodies.

 © 2019 The Authors
