# Supplementary material for: FAM83D directs protein kinase CK1α to the mitotic spindle for proper spindle positioning
Source: EMBO Rep. 2019 Jul 24;20(9):e47495. doi: 10.15252/embr.201847495 (PMC6726907; doi:10.15252/embr.201847495)
Supplement: Supplementary file 10 — Movie EV8 [file EMBR-20-e47495-s010.zip › Movie_EV8/Movie_EV8.docx]

**Movie EV8: *FAM83D* cDNA KI in *FAM83D^-/-^*) cell (clone 11) fails to divide along the hypotenuse of an L-shaped, fibronectin-coated micropattern.** A representative *FAM83D* cDNA KI in *FAM83D^-/-^* U2OS cell (clone 11) was imaged for 130 minutes with images captured at 10-minute intervals. Cell division is shown through time at 1 frame per second with the nucleus counterstained with Hoechst (shown in red), the L-shape micropattern and expected position of metaphase chromosomes overlaid in white dashed lines. The actual position of metaphase chromosomes is shown with a red line in the two frames prior to anaphase. Scale bar, 20 μm.
