# Supplementary figures and images for: FAM83D directs protein kinase CK1α to the mitotic spindle for proper spindle positioning
Source: EMBO Rep. 2019 Jul 24;20(9):e47495. doi: 10.15252/embr.201847495 (PMC6726907; doi:10.15252/embr.201847495)

**Figure 1A**

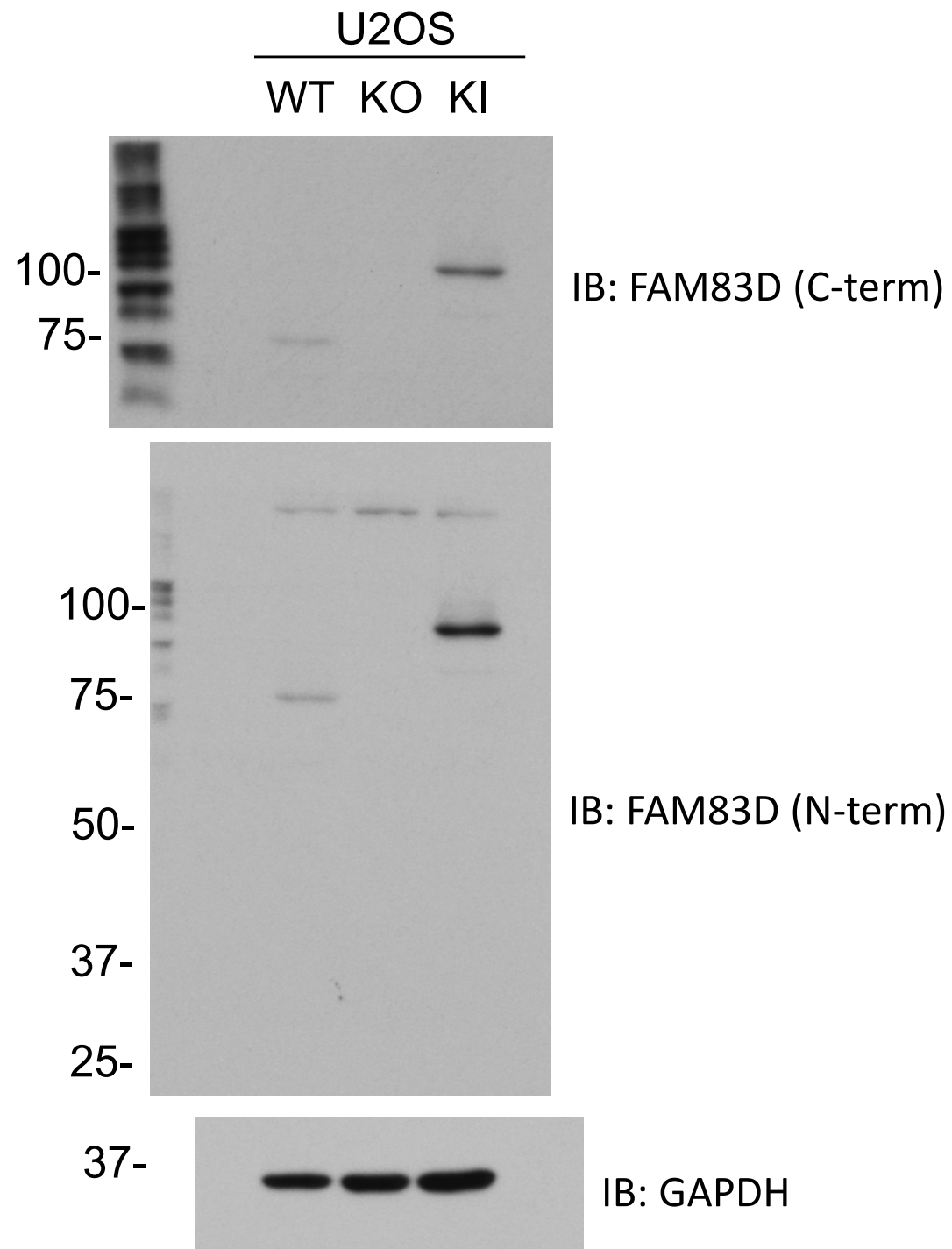

**Figure 1D**

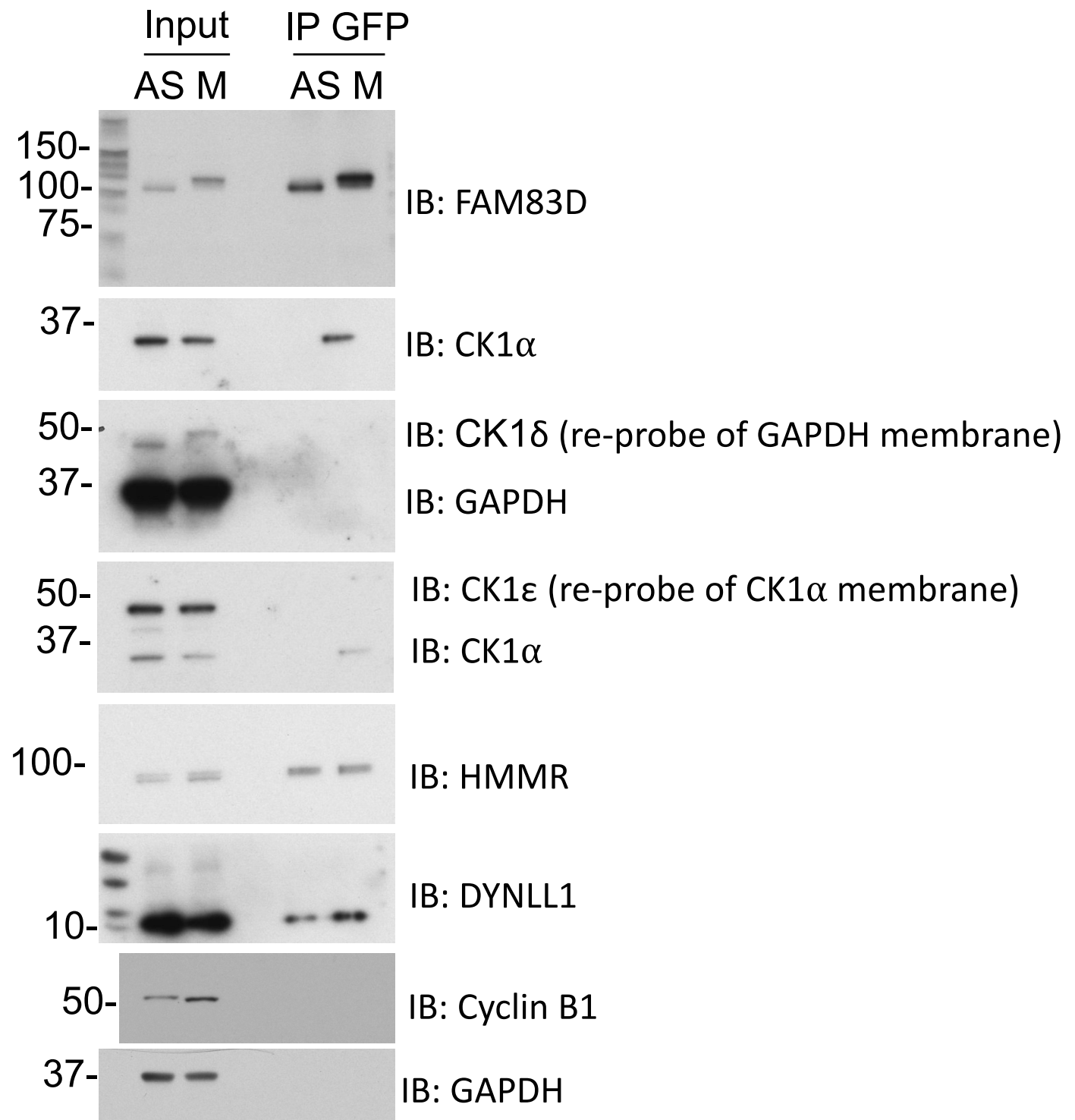

Figure 1E

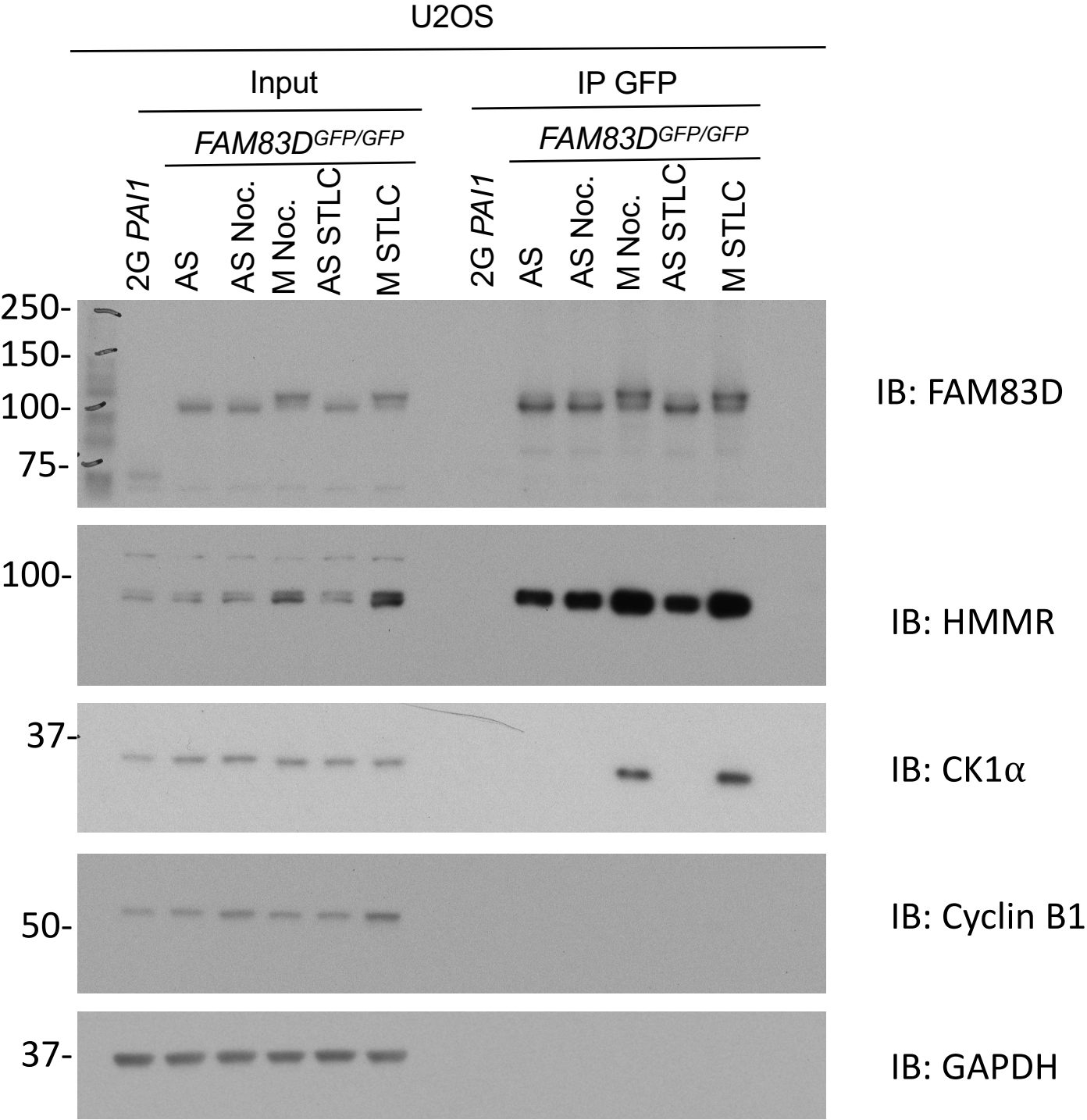

Figure 1G

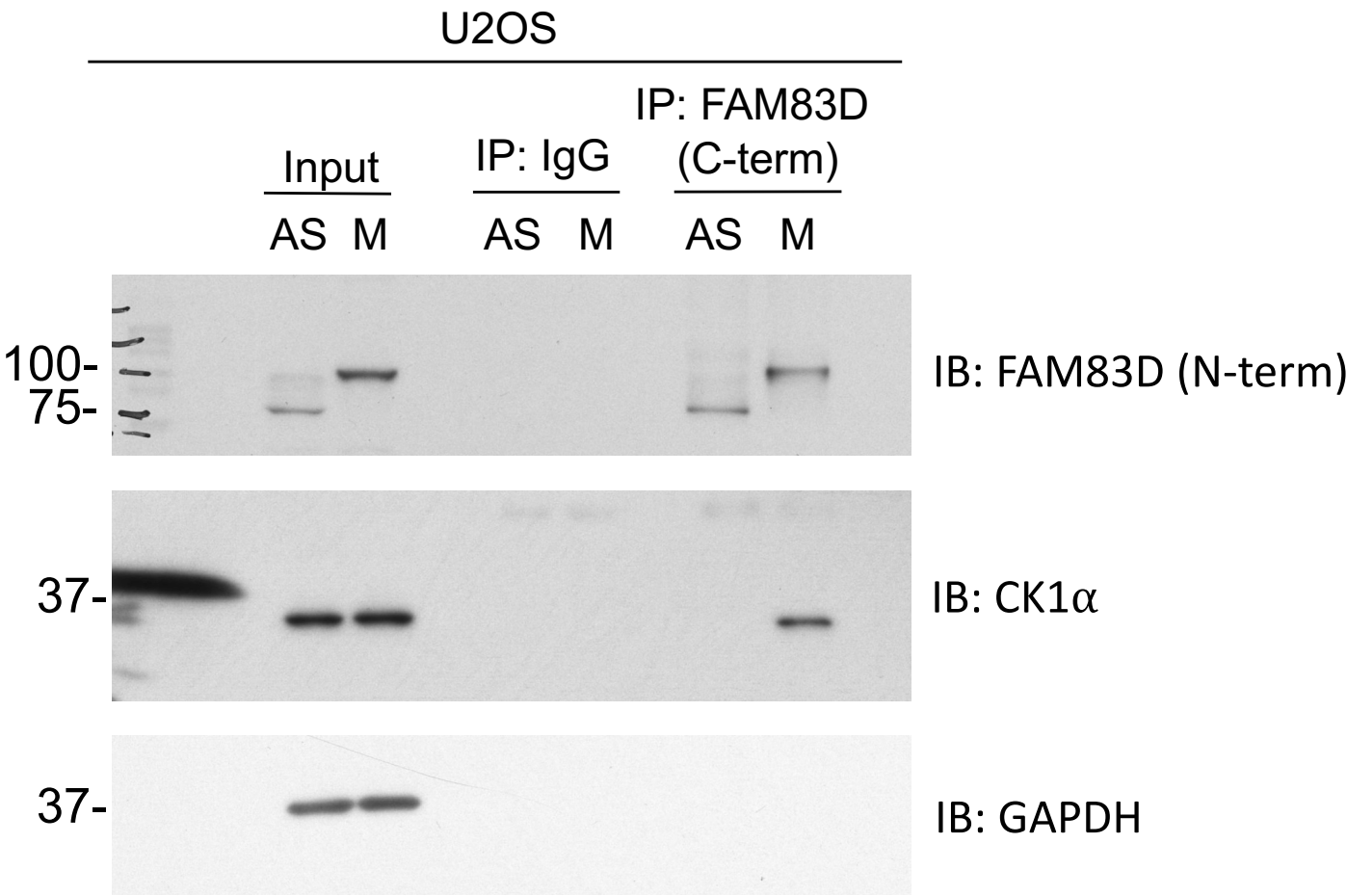

Figure 1H

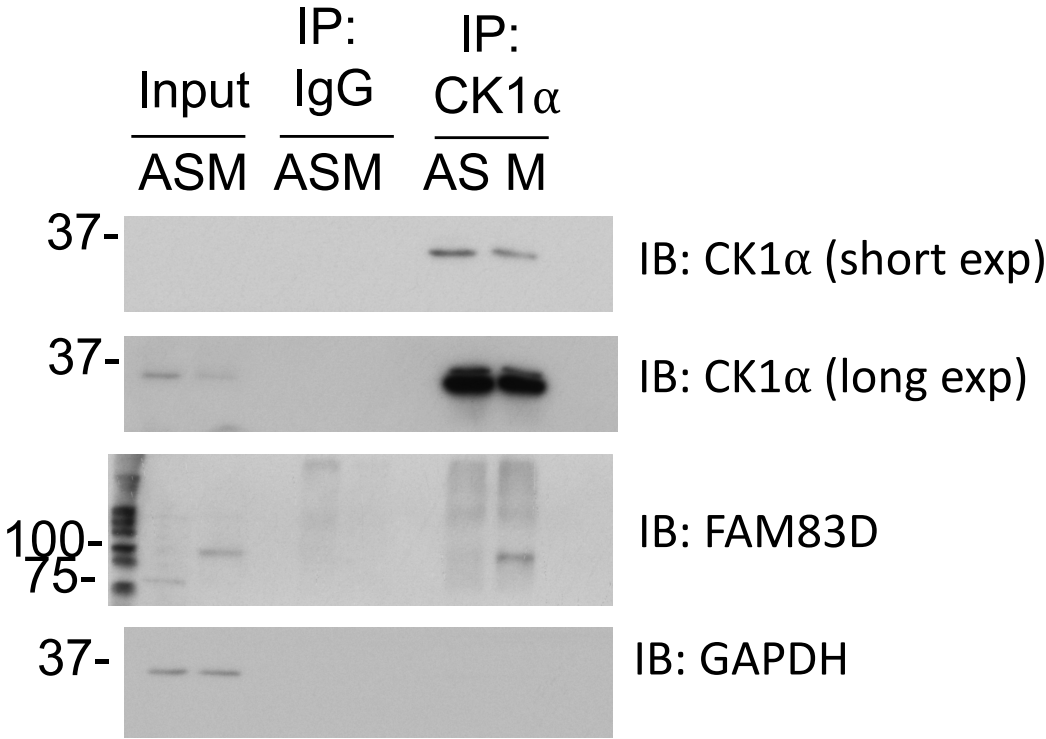

Figure 1I

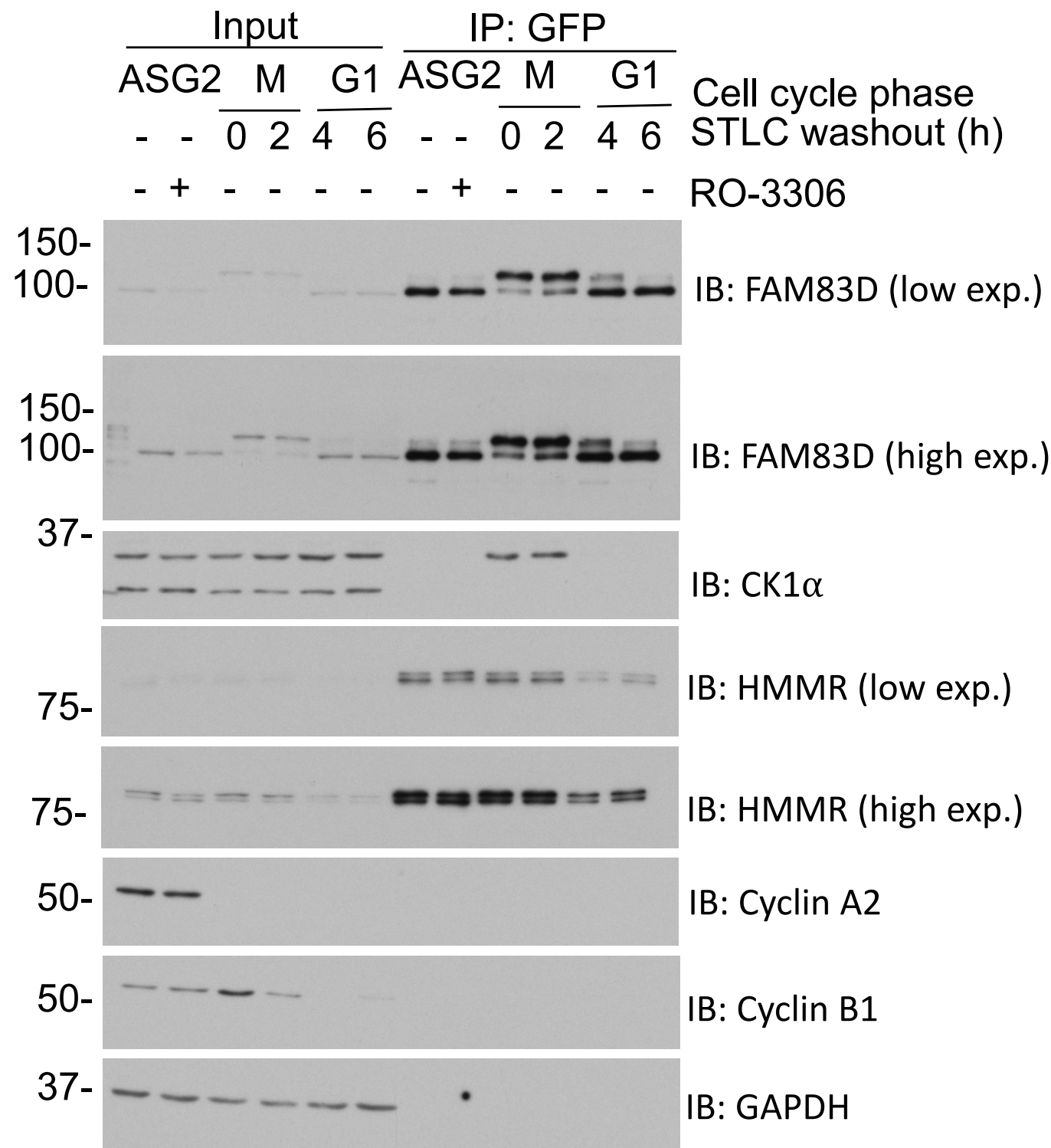

Figure 1K

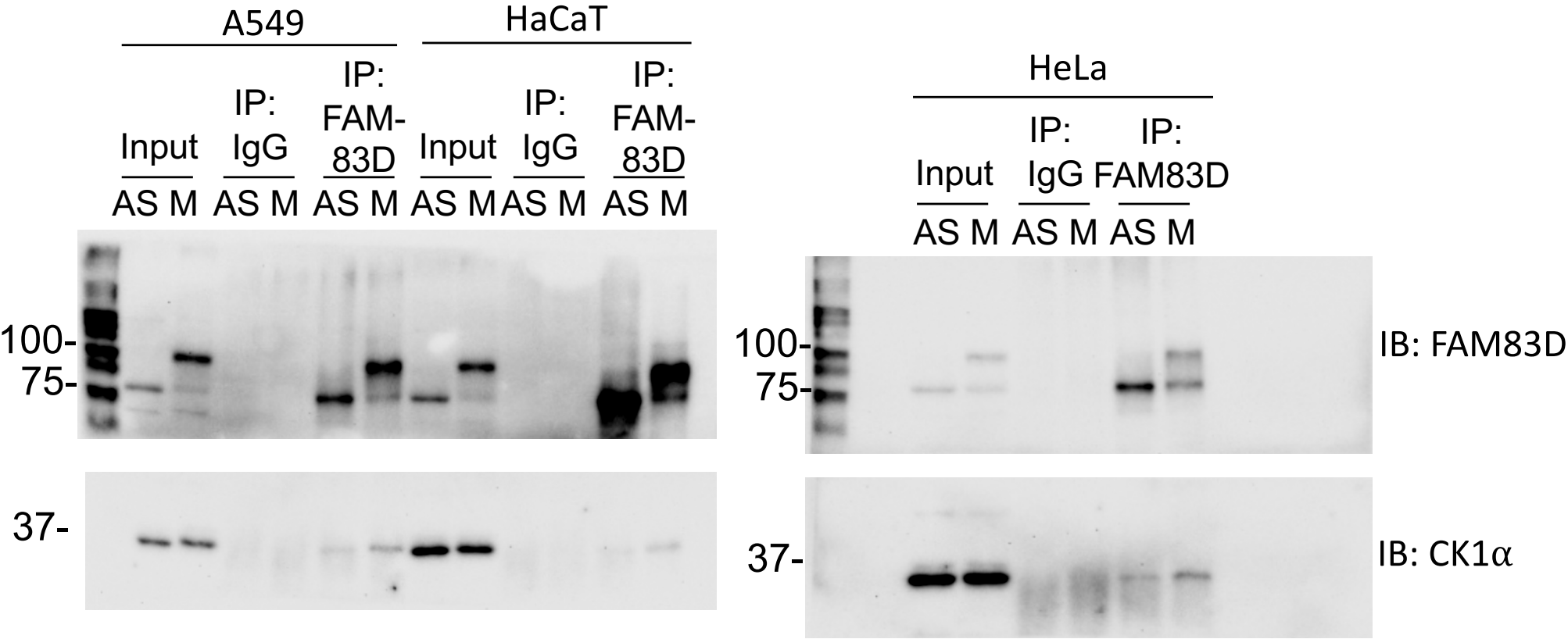

Supplement: Supplementary file 12 — Source Data for Figure 1 [file EMBR-20-e47495-s011.pdf]

**Figure 2D**

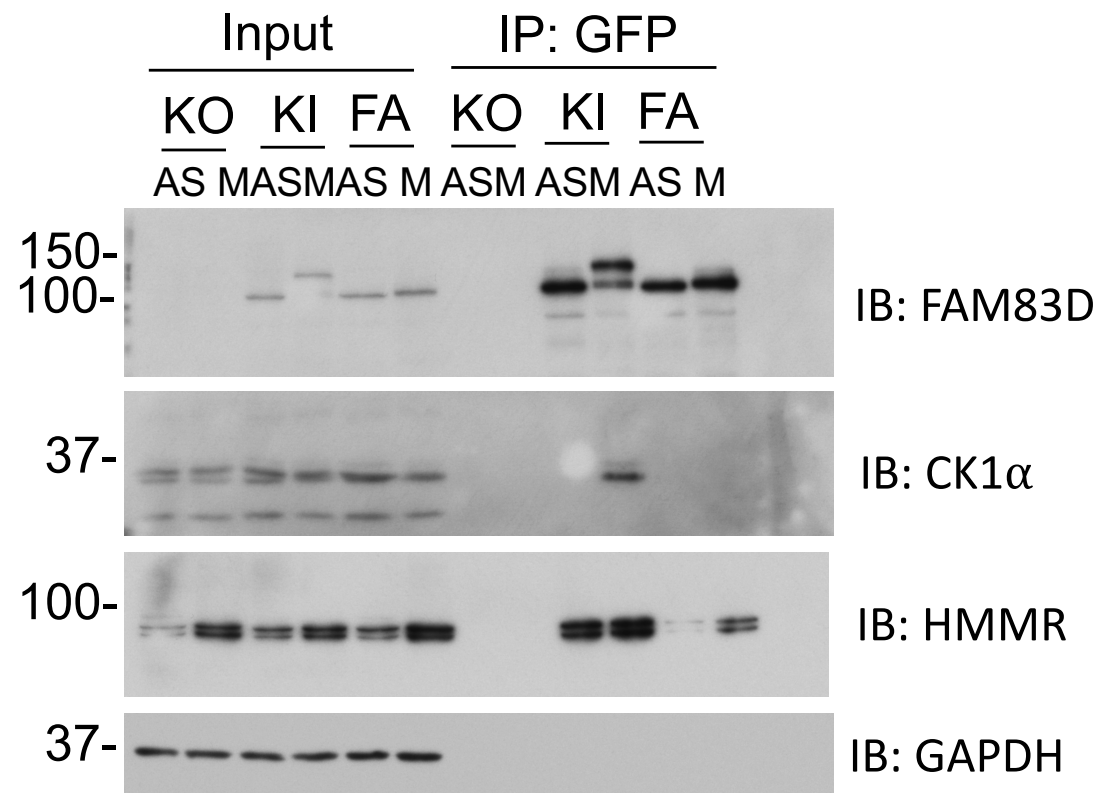

Figure 2F

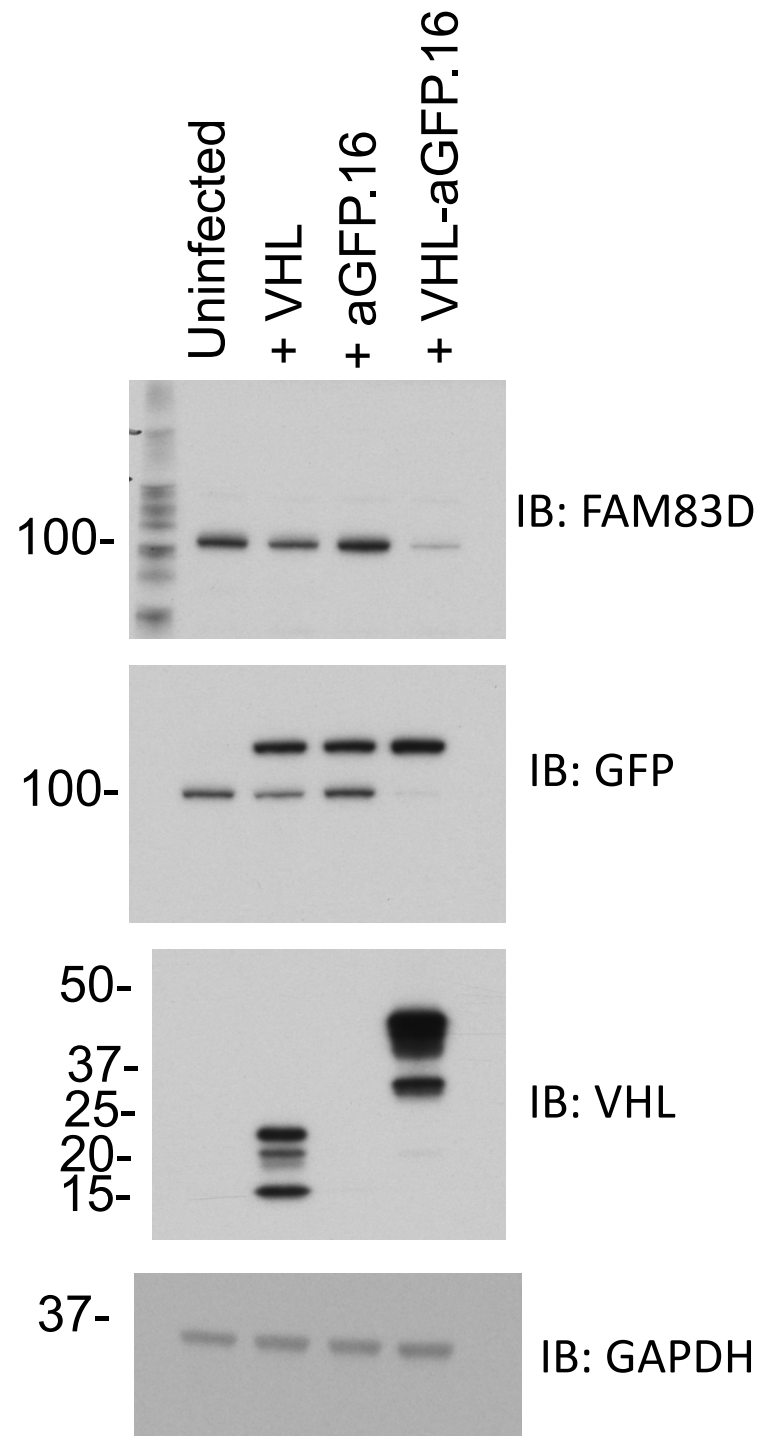

Supplement: Supplementary file 13 — Source Data for Figure 2 [file EMBR-20-e47495-s012.pdf]

Figure 3B

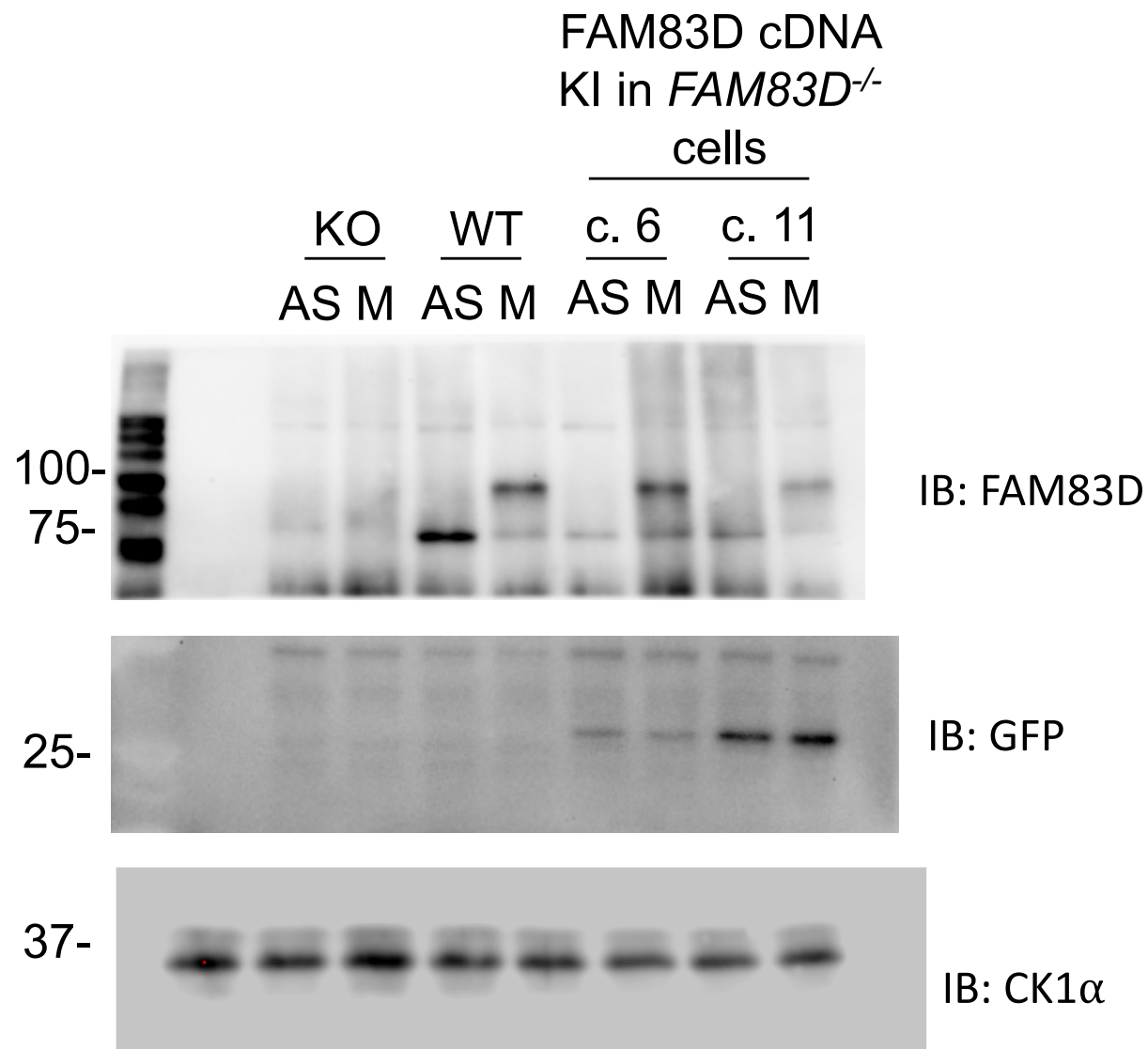

**Figure 3F**

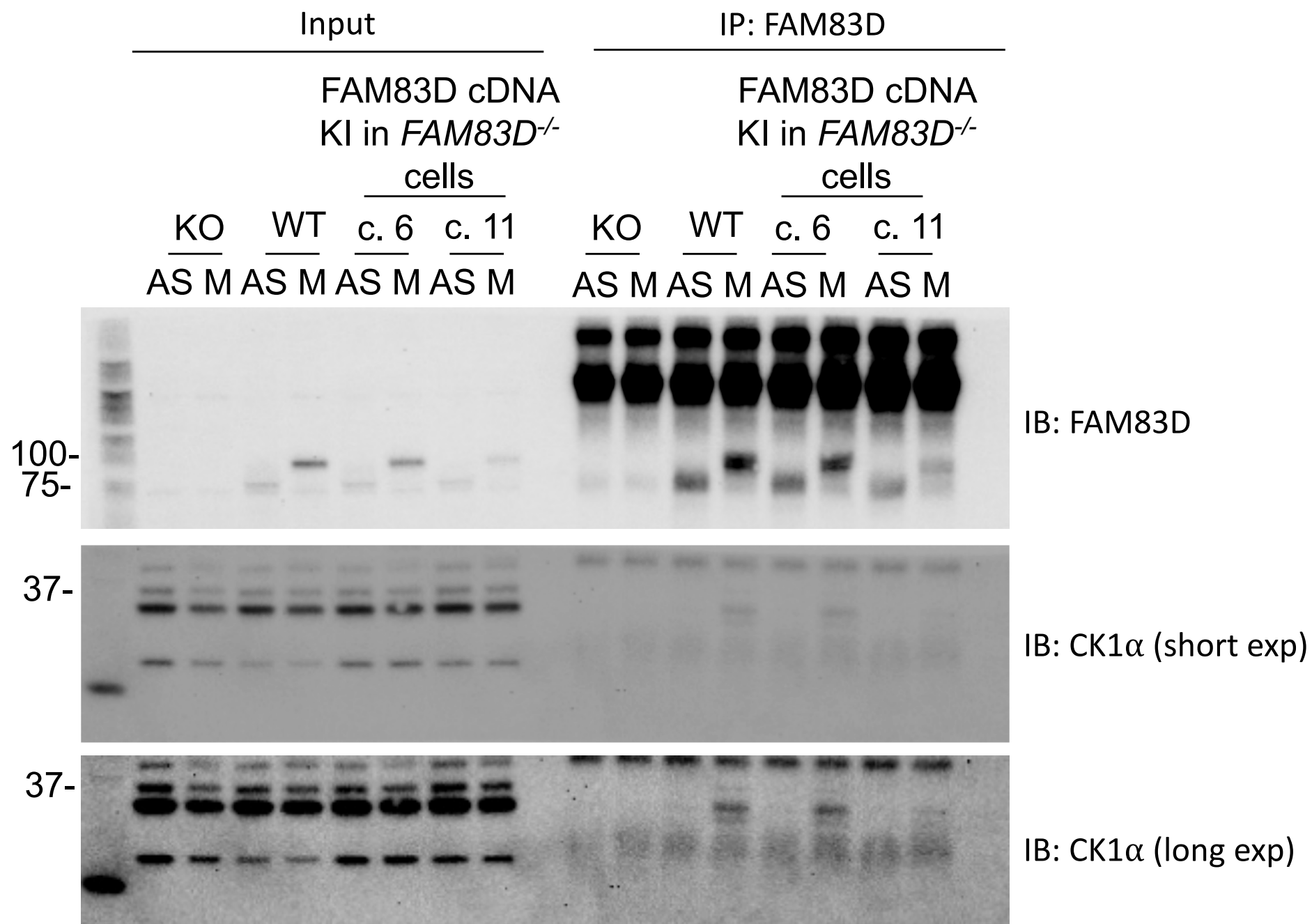

Supplement: Supplementary file 14 — Source Data for Figure 3 [file EMBR-20-e47495-s013.pdf]

Figure 4A

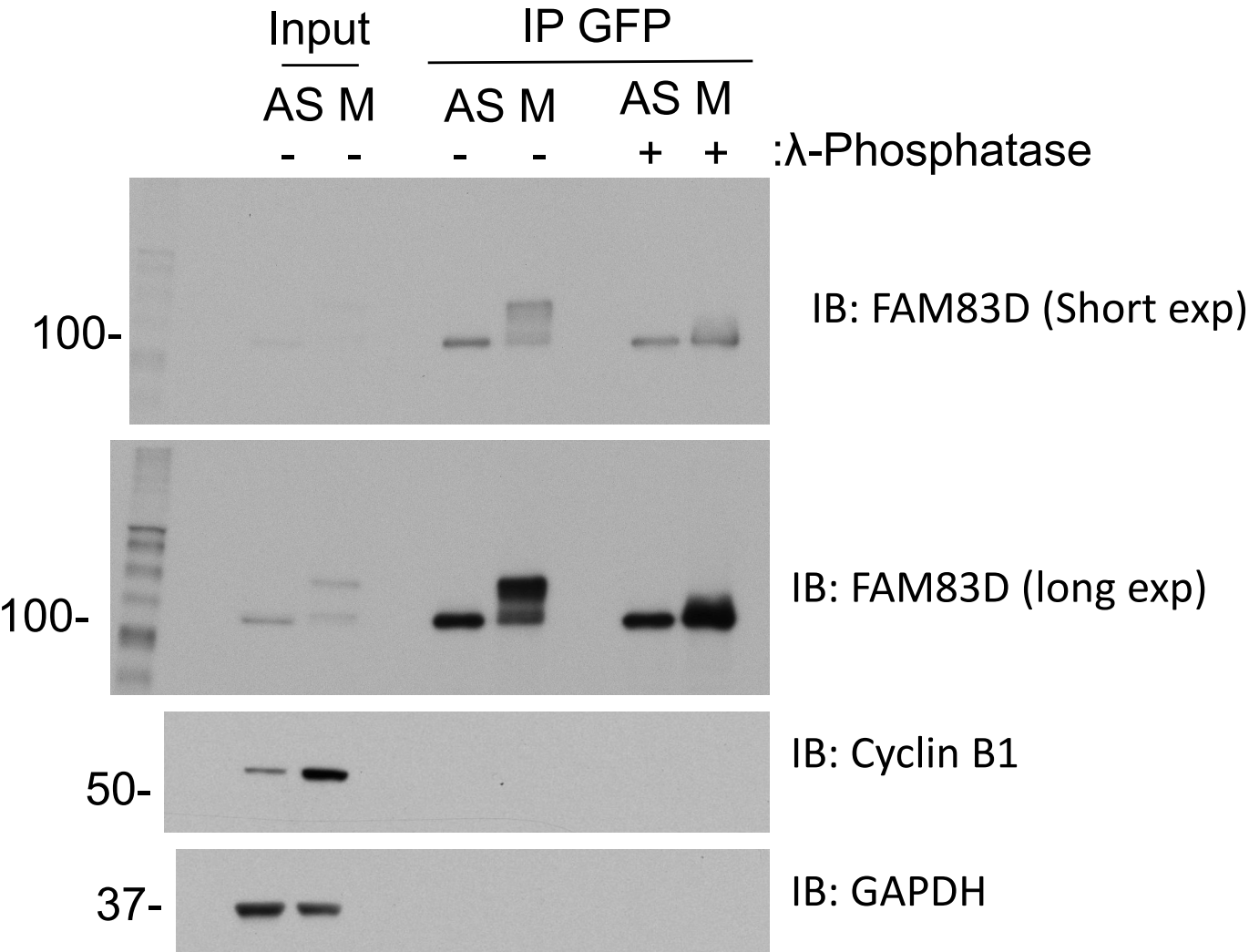



### Figure 4C

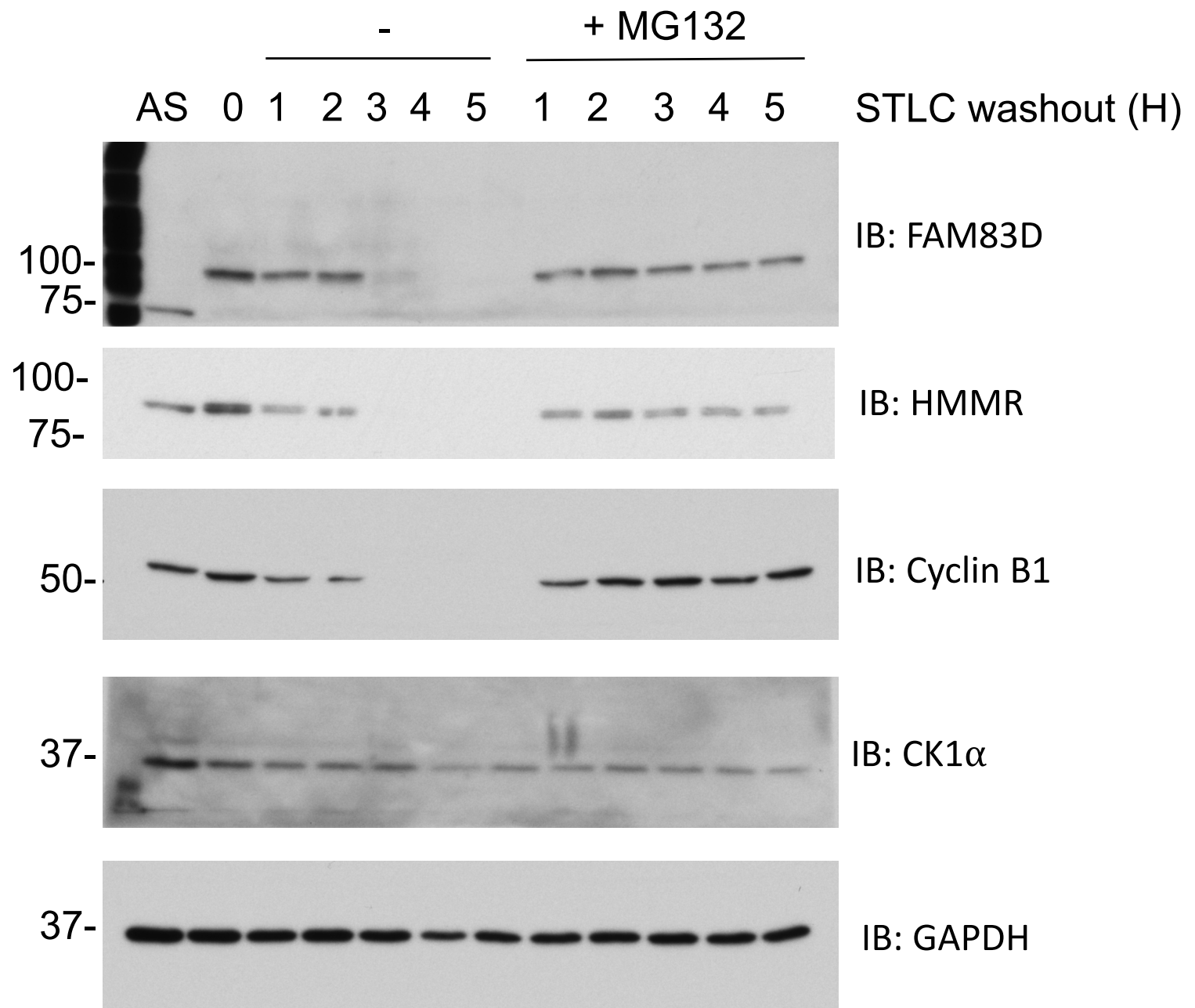

Figure 4D

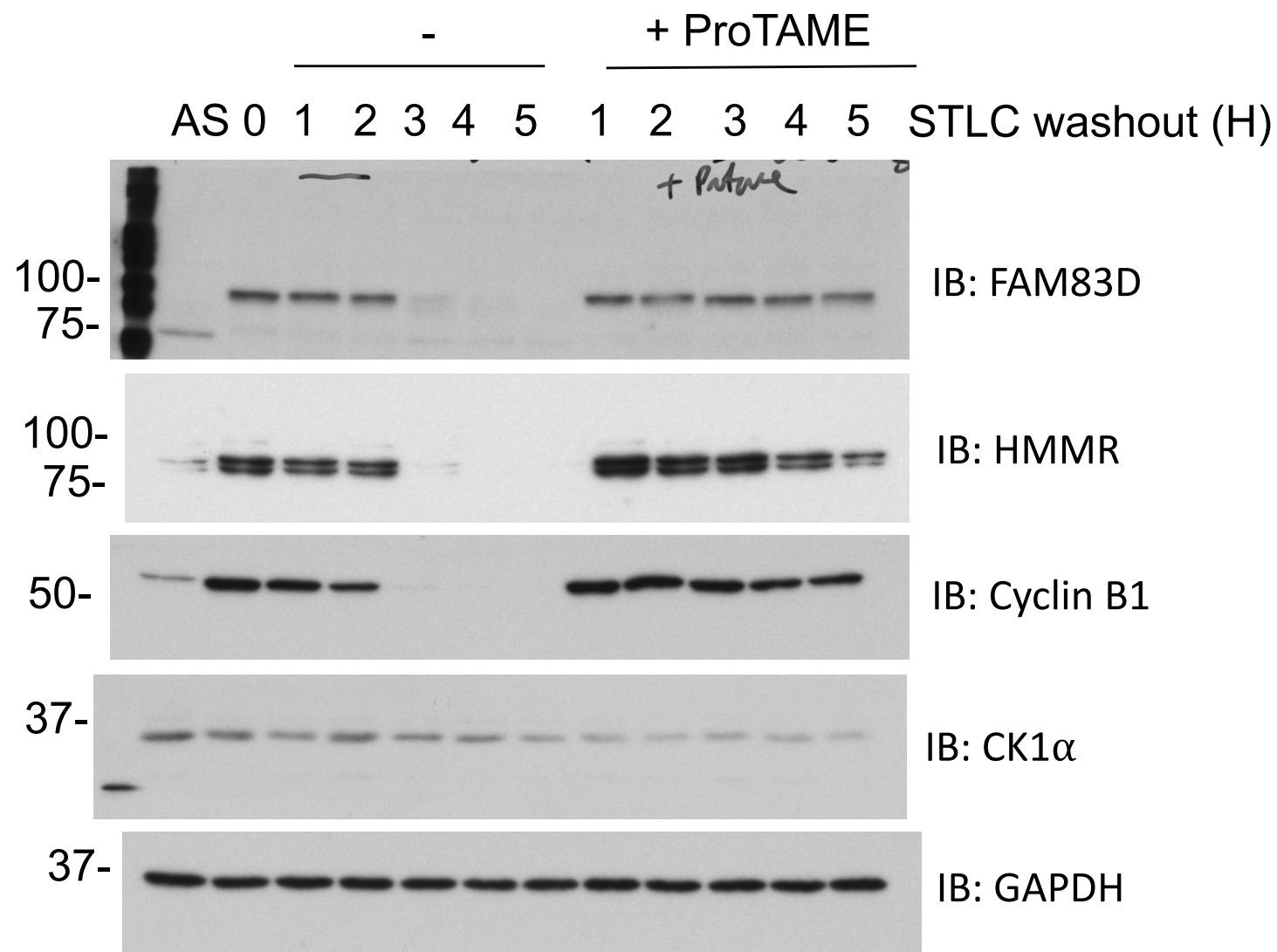

Figure 4E

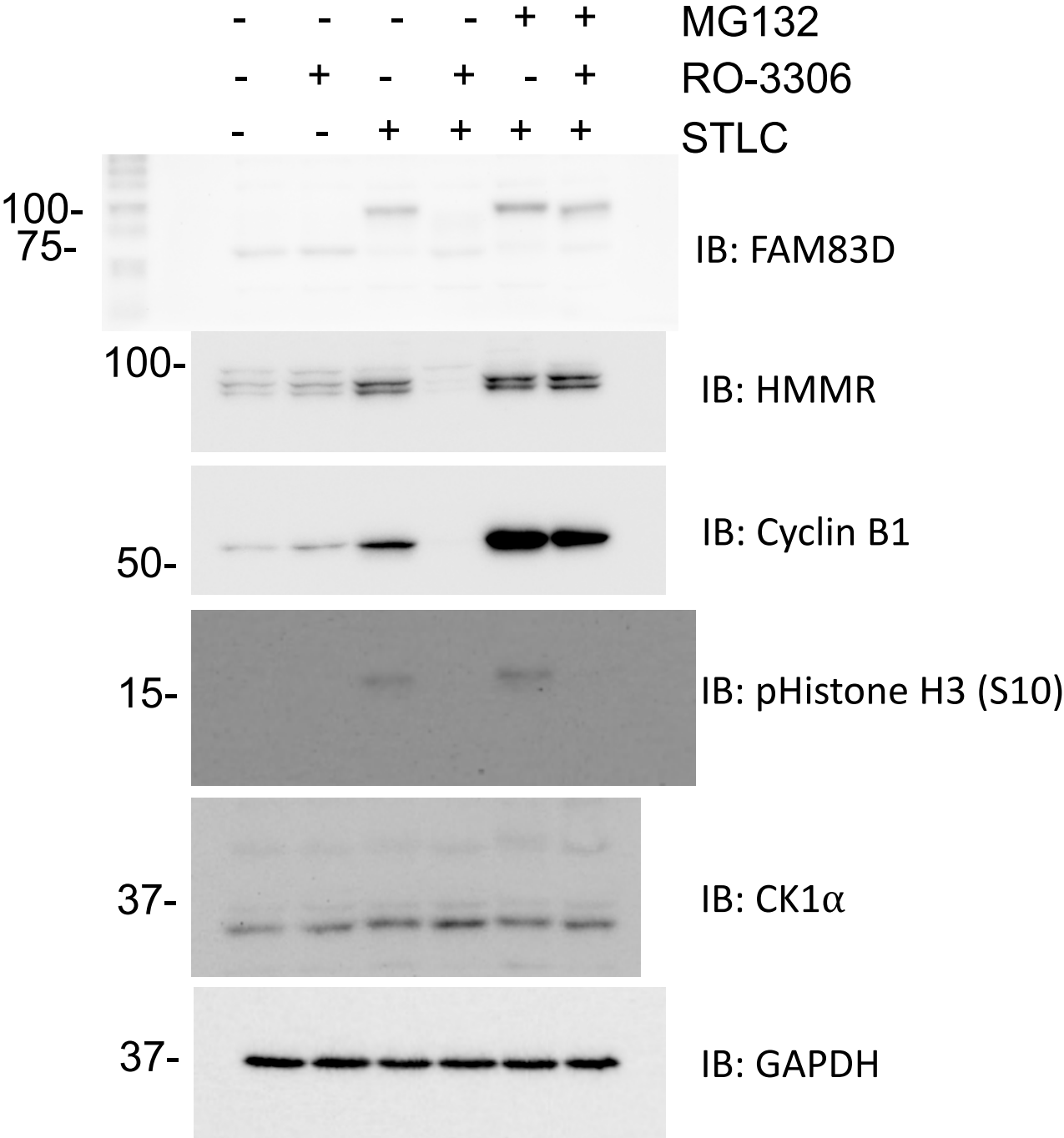

Figure 4H

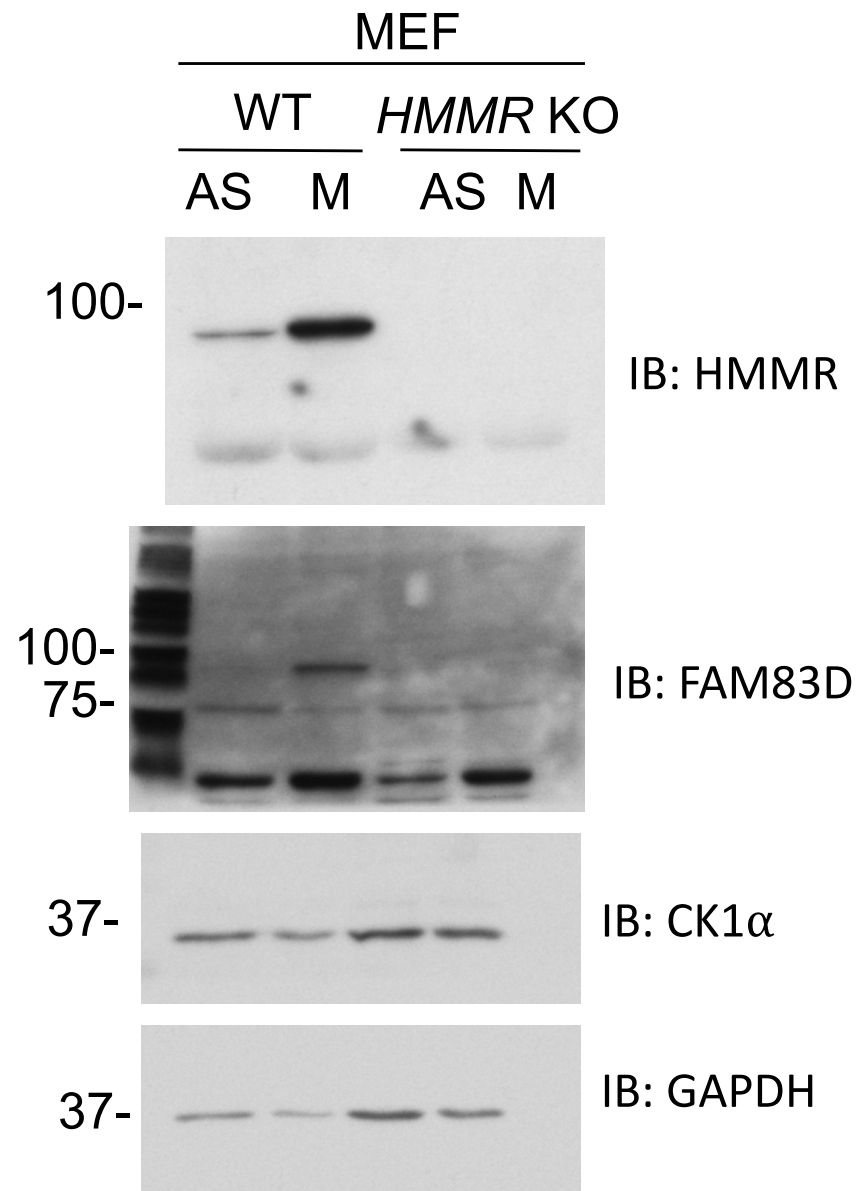

Supplement: Supplementary file 15 — Source Data for Figure 4 [file EMBR-20-e47495-s014.pdf]

**Figure 5B**

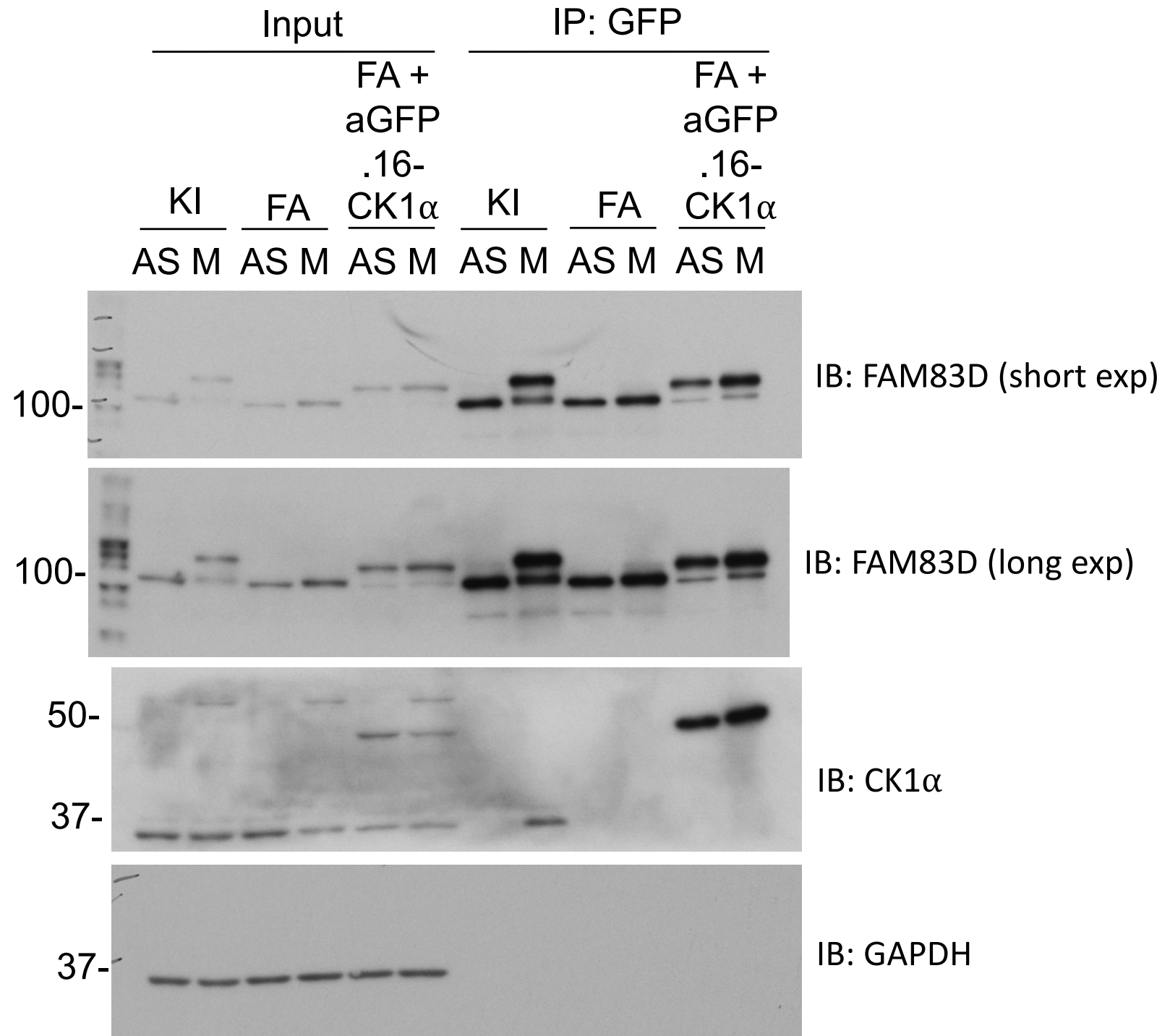

**Figure 5C**

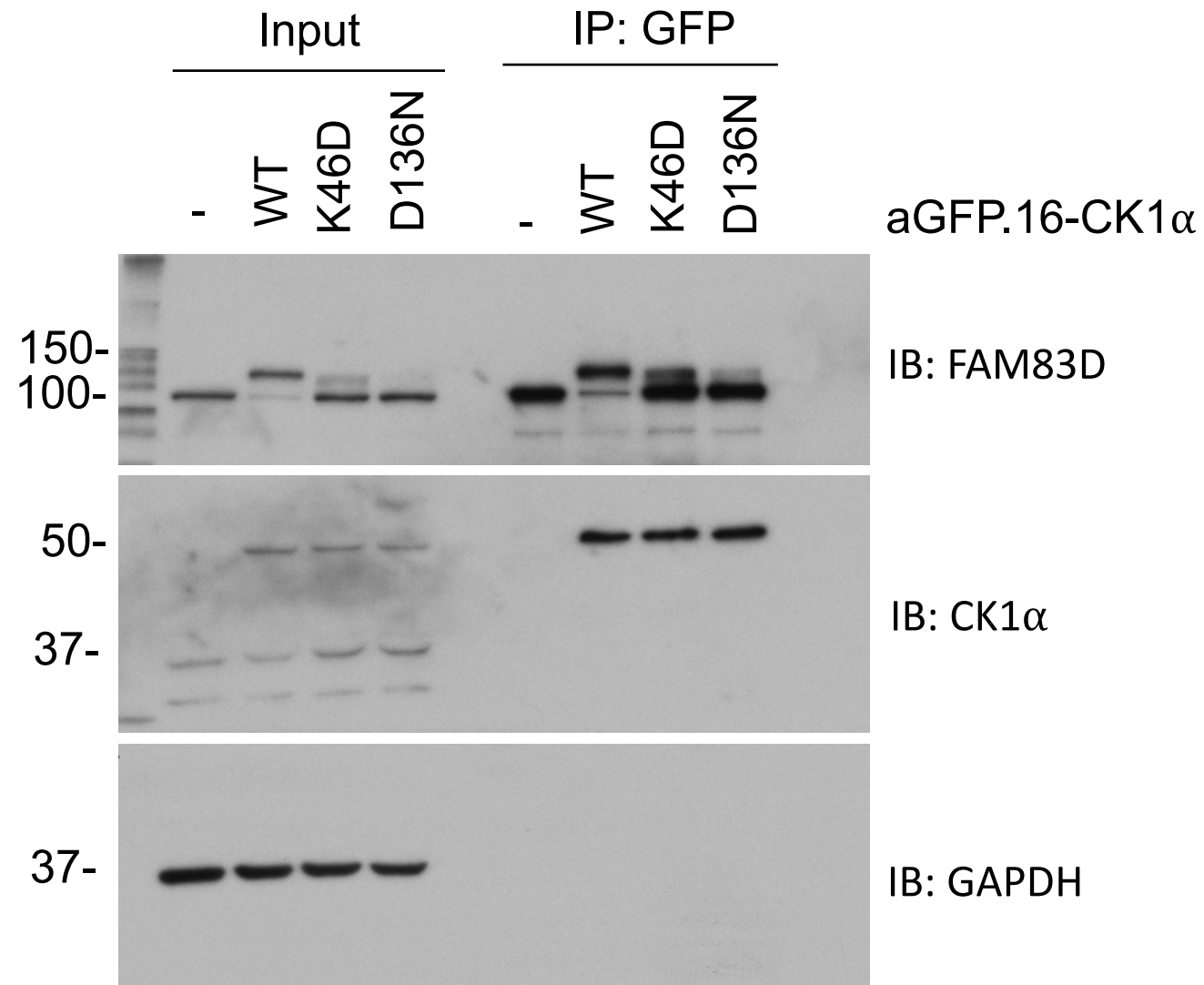

Figure 5F

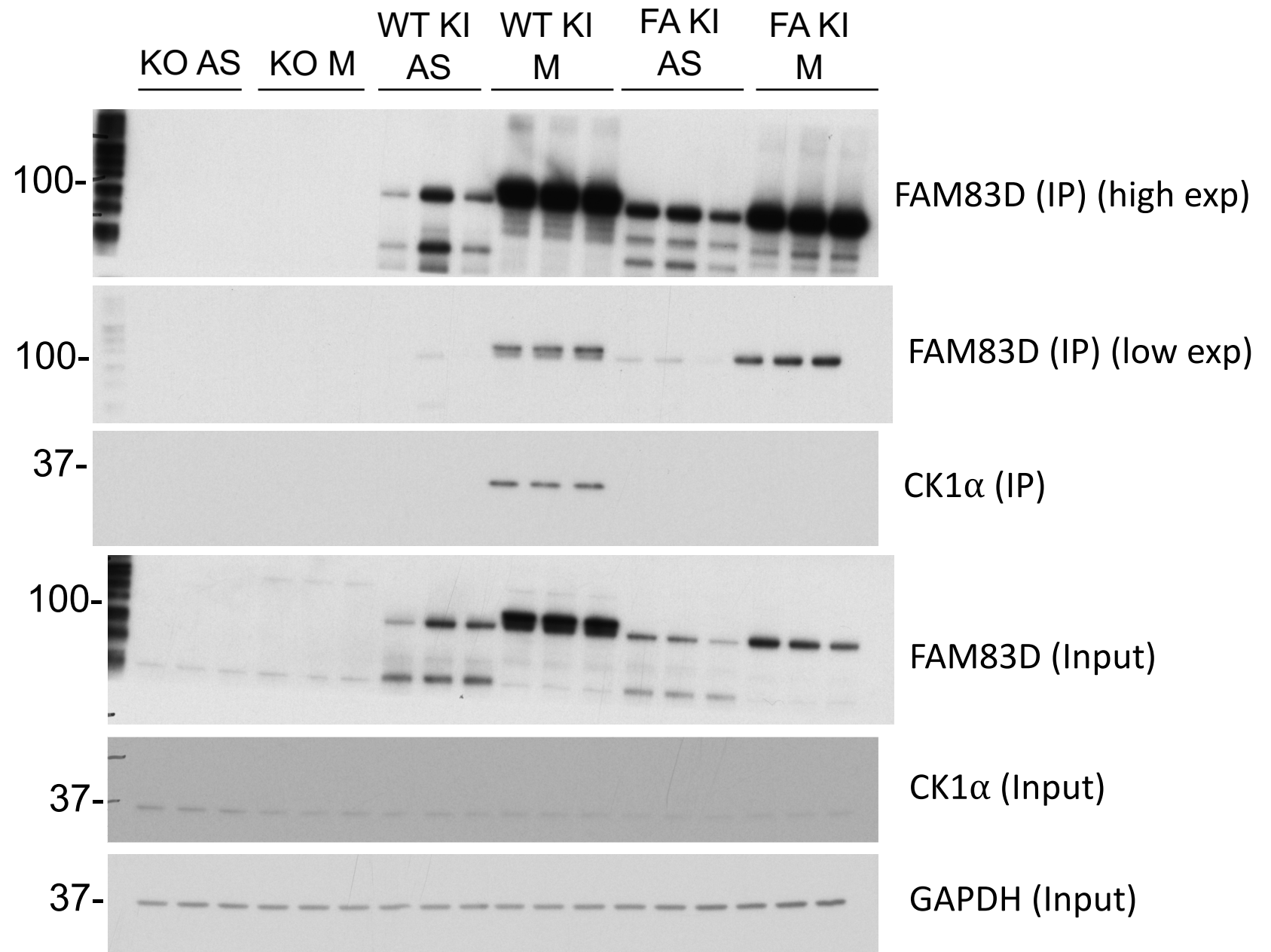

Supplement: Supplementary file 16 — Source Data for Figure 5 [file EMBR-20-e47495-s015.pdf]
